# Supplementary figures and images for: Auranofin and reactive oxygen species inhibit protein synthesis and regulate the level of the PLK1 protein in Ewing sarcoma cells
Source: Front Oncol. 2024 Jun 12;14:1394653. doi: 10.3389/fonc.2024.1394653 (PMC11199525; doi:10.3389/fonc.2024.1394653)

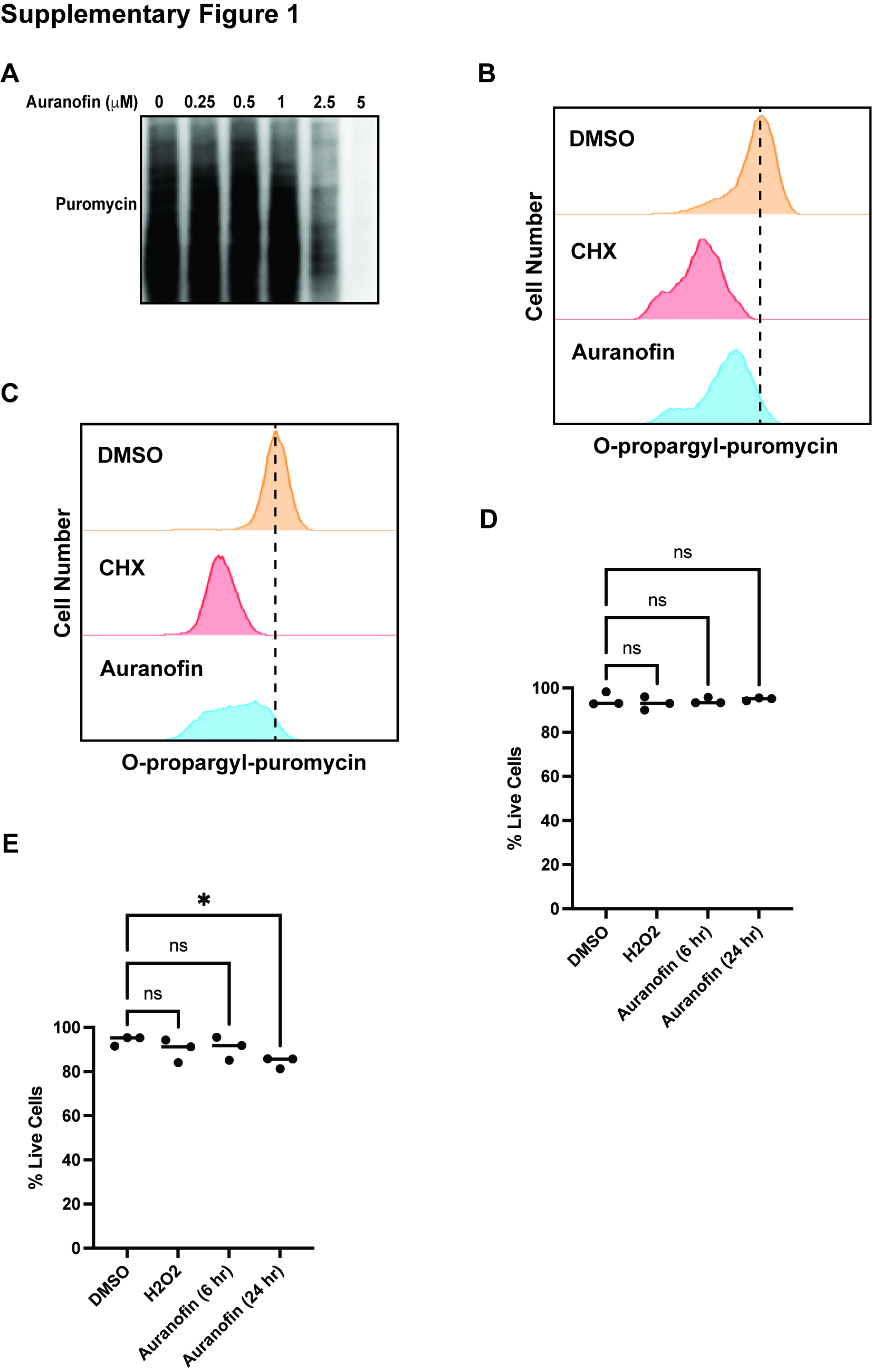

Supplement: Supplementary file 3 [file Image_1.tif]

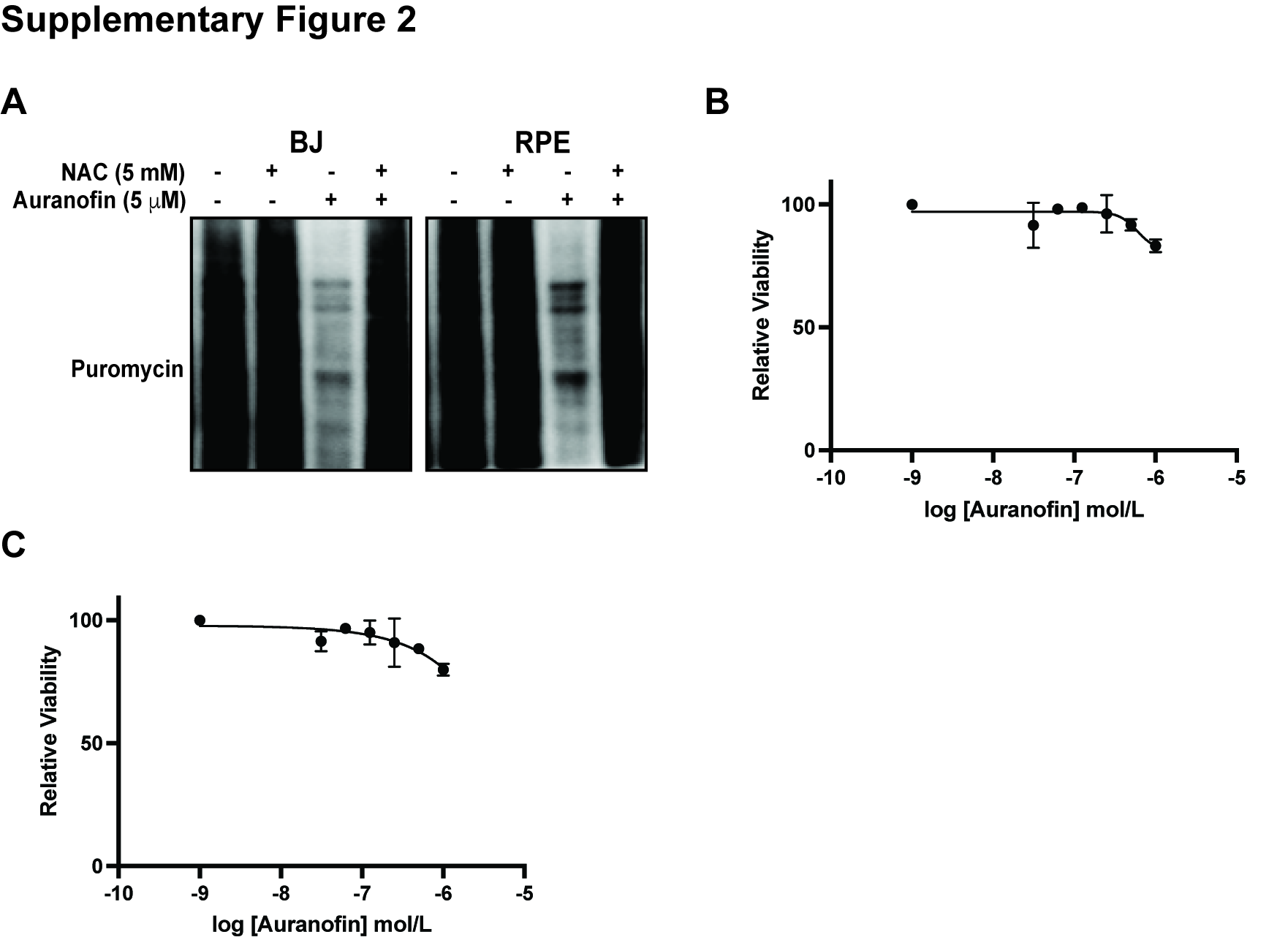

Supplement: Supplementary file 4 [file Image_2.tif]
